# Central hemodynamic parameters to predict cardiovascular outcomes and mortality among the elderly: protocol for a systematic review

**DOI:** 10.1590/1516-3180.2018.0209050618

**Published:** 2018-12-13

**Authors:** Grasiele Sausen, Tarsila Vieceli, Clarissa Garcia Rodrigues, Daniel Kipper, Airton Tetelbom Stein, Guilherme Brasil Grezzana

**Affiliations:** I PhD. Coordinator of the Experimental Research Section, Instituto de Cardiologia do Rio Grande do Sul, Fundação Universitária de Cardiologia (IC-FUC), Porto Alegre (RS), Brazil.; II MSc. Medical Student, School of Medicine, Universidade Federal de Ciências da Saúde de Porto Alegre (UFCSPA), Porto Alegre (RS), Brazil.; III PhD. Chief Executive Officer, Board of Directors, Global Research and Innovation Network (GRINN), Porto Alegre (RS), Brazil.; IV MD. Physician, Clínica Del Cuore, Antonio Prado (RS), Brazil.; V PhD. Provost of Research and Graduate Programs. Universidade Federal de Ciências da Saúde de Porto Alegre (UFCSPA), Porto Alegre (RS), Brazil.; VI PhD. Physician and Director, Clínica Del Cuore, Antonio Prado (RS), Brazil.

**Keywords:** Blood pressure, Hypertension, Aged, Mortality.

## Abstract

**BACKGROUND:**

Central blood pressure is a factor that may predict cardiovascular events. However, its use in clinical practice is not well consolidated. Therefore, the aim of our study will be to summarize the use of central hemodynamic parameters to predict cardiovascular-related outcomes and all-cause mortality.

**DESIGN AND SETTING:**

Protocol for systematic review of longitudinal observational studies conducted in healthcare institutions, as presented in the studies included.

**METHODS:**

We will perform a systematic search in the electronic databases MEDLINE (via PubMed), EMBASE and LILACS (via Virtual Health Library (VHL)), using health descriptors terms for elderly people and for hemodynamic indices of central blood pressure. We will include articles that evaluated hemodynamic indices and at least one of the following outcomes: all-cause mortality, total cardiovascular death, total non-cardiovascular death, myocardial infarction, stroke, coronary artery restenosis after percutaneous coronary intervention, revascularization and aortic syndromes. Two independent reviewers will conduct analysis on the abstracts selected and on the full-text articles. Two reviewers will independently perform data extraction and evaluate the methodological quality of the articles selected, and a third reviewer will evaluate any divergences. The methodological quality of the studies will be assessed in accordance with the ROBINS-I tool (Risk Of Bias In Non-randomized Studies of Interventions).

**RESULTS AND CONCLUSIONS:**

Through this systematic review, we intend to summarize evidence that supports the use of central hemodynamic parameters for central blood pressure to diagnose and perform prognostics on arterial hypertension in elderly patients within clinical practice and predict future cardiovascular events in this population.

**REGISTRATION:**

Prospero - CRD42018085264.

## INTRODUCTION

Brachial blood pressure (BP) is a parameter for predicting cardiovascular injury, morbidity and mortality^1^ that is widely used in clinical practice to assess cardiovascular risk. However, brachial BP does not correspond to the central BP that is assessed in the carotid artery and ascending aorta,^2^ which is an independent prediction factor for cardiovascular clinical events.^3^ Considering the aging process and the presence of some cardiovascular risk factors, the differences between central and brachial BP, and between central and wrist-assessed BP, tend to become smaller.^4^ However, central BP better reflects the pressure load associated with the left ventricle and coronary circulation and is therefore a potentially accurate risk marker and pressure target for efficiency assessments on therapeutic interventions.^5^ Moreover, the pharmacological superiority of vasodilator drugs for cardiovascular outcomes suggests that these will have a distinct effect on central BP, although the effects on brachial BP will be similar.^6^ Thus, this suggests that peripheral BP measurements are not a proper substitute for assessing the antihypertensive effects on arterial hemodynamics.^7^

Despite the applicability of central hemodynamic assessments, with diagnostic, therapeutic and prognostic scope, many aspects of these assessments continue to be pending matters. This is reflected in less widespread incorporation of this method into clinical practice. One of these issues is the lack of recommendations for use of central BP assessments to diagnose arterial hypertension within regular practice in the current guidelines. There are also deficiencies regarding standardization and validation for non-invasive central BP assessment instruments, and regarding the definitions for cutoff values for normal BP in different populations and among individuals of different ages.^8^ Furthermore, there is a need to clarify the reference interval for indirectly assessed central BP values, the age-related physiological increase in BP and the pathological increase in BP that relates to higher risk of cardiovascular disease.^8^

Lastly, the evidence available increasingly corroborates widespread usage of central BP assessment as a substitute tool for prediction of future cardiovascular events.^6^ Therefore, this topic deserves further investigation through the approach of conducting a systematic review of the literature.

## OBJECTIVES

Thus, the aim of the present protocol for a systematic review will be to summarize the evidence regarding the use of central hemodynamic parameters to predict cardiovascular-related outcomes such as total cardiovascular death, total non-cardiovascular death, myocardial infarction, stroke, coronary artery restenosis after percutaneous coronary intervention, revascularization and/or aortic syndromes, and all-cause mortality.

## METHODS

### Protocol and registration

This systematic review will be reported in accordance with the guidelines of the Meta-Analysis Of Observational Studies in Epidemiology (MOOSE) group.^9^ It has been registered in the International Prospective Register of Systematic Reviews (Prospero) database,^10^ under the code CRD42018085264.

### Eligibility criteria

We will include full peer-reviewed publications from longitudinal observational comparative studies, in which patients aged 60 years or older were included (we will consider the mean age presented in the study). The studies included need to report at least one of the following indexes of central hemodynamics: central systolic blood pressure (SBP), central pulse pressure (PP), central augmentation index (AIx), aortic pressure, wave reflections (WR), pulse wave velocity (PWV) and/or carotid systolic blood pressure (CSBP). Additionally, these studies need to report at least one of the following outcomes: total (all-cause) mortality, total cardiovascular death, total non-cardiovascular death, myocardial infarction, stroke, coronary artery restenosis after percutaneous coronary intervention, revascularization and/or aortic syndromes. We will exclude studies if they reported results from duplicate populations.

### Information sources

We will search the following electronic databases: MEDLINE (via PubMed), EMBASE (via Elsevier) and Virtual Health Library (VHL). This last platform contain citations from LILACS (Literatura Latino-Americana e do Caribe em Ciências da Saúde), IBECS (Índice Bibliográfico Español en Ciencias de la Salud), MEDLINE, Cochrane Library and SciELO. In addition, we will manually search the references of the articles included and we will perform a citation analysis on the studies included, using Google Scholar. Gray literature will not be searched. We will also ask for experts’ suggestions, through email communications.

### Search

The initial search will comprise the Mesh terms “Aged”, “Aged, 80 and over”, “Pulse wave analysis” and related entry terms; other terms relating to central hemodynamics such as “Central systolic blood pressure”, “Central pulse pressure”, “Central augmentation index”, “Central pressures”, “Aortic pressure” and “Wave reflections”; and a sensitive search strategy for observational studies.

### Study selection

The titles and abstracts of the articles retrieved will be independently evaluated by two reviewers (GS and TV). Abstracts that do not provide enough information regarding the eligibility criteria will be kept for full-text evaluation. The reviewers will independently evaluate full-text articles and determine study eligibility. Any disagreements will be resolved by reaching a consensus and, if disagreement persists, these two reviewers will ask a third reviewer for an opinion (GG).

### Quality of studies

Risk of bias will be evaluated by ranking each study in accordance with the ROBINS-I tool (Risk Of Bias In Non-randomized Studies of Intervention).^11^ The following types of bias will be considered: bias due to confounding, bias in selecting participants for the study, bias in classifying interventions, bias due to deviations from intended interventions, bias due to missing data, bias in measuring outcomes, bias in selecting the reported result and overall bias. Each item will be classified as presenting low, moderate, serious or critical risk of bias; or as presenting “no information” when the article provides no information on which to base a judgement about the risk of bias for this domain.

### Appraisal of uncertainty of the evidence

GRADEpro GDT^12^ will be used for appraisal of uncertainty of the evidence from the studies included. Tables summarizing the findings will be built in order to grade the evidence available. In relation to each outcome, these factors will include risk of bias, inconsistency (heterogeneity), indirectness, imprecision, and bias of publication. Each type of evidence will be graded as very low, low, moderate or high.

### Data extraction

Two reviewers (GS and TV) will independently conduct data extraction and any disagreements will be resolved by bringing in a third reviewer (GG). The general characteristics of the studies will be noted, such as: study title, author, journal and year of publication, study design, inclusion and exclusion criteria, outcome definitions, outcome measurements and follow-up. In addition, we will extract specific information about indexes of central hemodynamics and their predictive values (when available).

### Data analysis

Descriptive analysis will be performed on the studies, including study characteristics and main results. We plan on performing meta-analyses, if appropriate. The risk estimates for each study will be reported as hazard ratios, relative risks (RRs) or odds ratios. Hazard ratios will be treated in the same way as RRs. Adjusted risk estimates from multivariate models will be used to control for possible selection bias in the original studies. Heterogeneity across studies will be quantified by means of the I² statistic, and the random-effects model will be used to obtain the pooled RRs. The RRs and confidence intervals (CIs) of comparable studies will be illustrated using forest plots. We will generate funnel plots to assess the presence of publication bias, and Duval and Tweedie’s trim-and-fill method will be used to assess the implications of publication bias. Results will be considered statistically significant at P < 0.05. All analyses will be performed using R language.

## DISCUSSION

Evaluation of central hemodynamic measurements may be an effective way to obtain an accurate diagnosis of arterial hypertension and, consequently, may lead to appropriate therapeutic and prognostic decisions. There is an independent relationship between central pressure findings and future cardiovascular events, independently of assessments on peripheral BP, including in elderly populations with coronary arterial and chronic renal disease.^6^ Thus, the main potential of the present study is that, through a large and systematic review of the literature, it may bring to light the current evidence supporting the use of central BP parameters as a constant practice for diagnostics, therapeutics and prognostic evaluation in the context of arterial hypertension.

The main limitation of the present study will be that it uses the findings of diagnostic, therapeutic and prognostic evidence from longitudinal studies, to the detriment of more robust studies for supporting broad indication of evaluation of central BP parameters in the context of arterial hypertension. Another limitation to be considered is the fact that the present study proposes to evaluate the central hemodynamic parameters for BP in elderly populations. This aspect of the study design therefore does not allow us to evaluate the pathological condition of systolic arterial hypertension in isolation, in younger individuals.

Lastly, choosing a tool to assess risk of bias was a challenging task, given that different tools can lead to different results.^13^ We acknowledge that the ROBINS-I is an instrument primarily built to assess risk of bias of non-randomized studies of interventions, which is not the case of our study. However, it assesses a causal relationship and, thus, it can be used to assess risk of bias of other type of study designs as well. We accept that ROBINS-E (Risk Of Bias In Non-Randomized Studies of Exposures)^14^ would potentially be the ideal tool; however, this tool is still under development and therefore we did not consider using it in our study since it has not been validated yet. We considered using the NOS (Newcastle-Ottawa Scale) because we have experience of using this instrument in previous studies. However, it has been well reported that NOS has several limitations including low reliability between individual reviewers and lack of evidence that NOS can identify studies with biased results, which underscores the need for revisions and/or more detailed guidance for systematic reviewers.^15^^,^^16^^,^^17^ In this light, even though ROBINS-I was primarily developed for intervention studies, we considered it to be the best available option for assessing risk of bias in our study.

## CONCLUSION

Through this systematic review, we intend to summarize evidence that supports the use of central hemodynamic parameters for central blood pressure to diagnose and perform prognostics on arterial hypertension in elderly patients within clinical practice and predict future cardiovascular events in this population.
